# Comparison of Enzootic Risk Measures for Predicting West Nile Disease, Los Angeles, California, USA, 2004–2010

**DOI:** 10.3201/eid1808.111558

**Published:** 2012-08

**Authors:** Jennifer L. Kwan, Bborie K. Park, Tim E. Carpenter, Van Ngo, Rachel Civen, William K. Reisen

**Affiliations:** University of California, Davis, California, USA (J.L. Kwan, B.K. Park, T.E. Carpenter, W.K. Reisen);; and Los Angeles County Department of Public Health, Los Angeles, California, USA (V. Ngo, R. Civen)

**Keywords:** West Nile virus, Culex pipiens quinquefasciatus, risk assessment, Mantel-Haentzel relative risk, Los Angeles, area under the curve, AUC, receiver-operating characteristic curve, ROC curve, viruses, zoonoses, California, vector-borne infections

## Abstract

In Los Angeles, California, USA, 2 epidemics of West Nile virus (WNV) disease have occurred since WNV was recognized in 2003. To assess which measure of risk was most predictive of human cases, we compared 3 measures: the California Mosquito-Borne Virus Surveillance and Response Plan Assessment, the vector index, and the Dynamic Continuous-Area Space-Time system. A case–crossover study was performed by using symptom onset dates from 384 persons with WNV infection to determine their relative environmental exposure to high-risk conditions as measured by each method. Receiver-operating characteristic plots determined thresholds for each model, and the area under the curve was used to compare methods. We found that the best risk assessment model for human WNV cases included surveillance data from avian, mosquito, and climate sources.

## Abstract 2

The best model comprised enzootic surveillance data from avian, mosquito, and climate sources.

West Nile virus (WNV; family *Flaviviridae*, genus *Flavivirus*) is amplified within a mosquito–bird cycle, with tangential transmission to equids and humans (*1*). Since the introduction of WNV into Los Angeles, California, USA, in 2003, our research (*2**–**5*) has focused on surveillance indicators for enzootic WNV transmission and prediction of human cases. The Greater Los Angeles County Vector Control District (GLACVCD) serves >6 million of the ≈10 million residents of Los Angeles County and conducts year-round surveillance for WNV activity (*6*). In addition to having a robust surveillance dataset, Los Angeles County is a suitable location for evaluating environmental risk because the large human population enables the sensitive detection of dead birds (*7*), increases opportunities for human–vector contact, and experienced 2 outbreaks during the study period (*6*).

We compared the predictive ability of 3 measures of human risk by using time-series graphs, sensitivity, specificity, positive predictive value (PPV), and concordance between human case onset and states of high risk based on enzootic transmission during 2004–2010. We believed that for operational decision support a successful risk measure should correctly 1) identify periods of low risk when few or no cases occur, 2) predict high or increased risk before human cases occur, and 3) identify periods of high risk concurrent with the occurrence of human cases_._

The 3 measures of risk we compared were the California Mosquito-Borne Virus Risk Assessment (CMVRA), the vector index, and the Dynamic Continuous-Area Space-Time (DYCAST) system. The CMVRA (*8*) calculates risk on the basis of ranks of environmental variables for enzootic transmission and is used by health agencies throughout California to measure risk. At its inception, the CMVRA was evaluated retrospectively for its ability to detect cases of Western equine encephalomyelitis virus (family *Togaviridae*, genus *Alphavirus*) and St. Louis encephalitis virus (family *Flaviviridae*, genus *Flavivirus*) in California during low-, medium- and high-risk seasons (*9*). Additional assessment of the ability of CMVRA to track WNV cases in Bakersfield, California, produced impressive results during 2004 and 2007 (*10**,**11*).

The second method was the vector index, an estimate of the number of infected mosquitoes collected per trap-night. This index successfully determined human risk in Colorado (*12**,**13*) and is used by the Colorado Department of Public Health and Environment (www.cdphe.state.co.us/dc/zoonosis/wnv/wnvsentinel.html).

The third method was the DYCAST (*14*) system, which provides an assessment of risk in time and space by using reports of dead birds from the California Department of Public Health Dead Bird Hotline. This risk estimate differs from the previous 2 in that the spatial scale is fine (0.44 km^2^ grid cells), it is computationally more complex, and it does not rely on laboratory test results (*15*).

Understanding the characteristics of risk estimates to determine the best predictive measure for human cases is needed for several reasons. First, reducing the rate of false-positive results will reduce message fatigue associated with repeated false warnings of high-risk conditions. Second, increasing the proportion of high-risk areas correctly identified (sensitivity) can reduce the costs associated with emergency mosquito control by correctly focusing timely intervention. Third, a qualitative assessment of risk estimates that incorporates different variables for enzootic transmission enables understanding of the ability of different assemblages of surveillance data for predicting human risk. Overall, a better understanding of the tools used in decision support for emergency intervention can only improve the protection of human health.

## Materials and Methods

The epidemiology of WNV in Los Angeles has been described in detail (*6*). Methods used for data collection for each risk assessment tool are summarized briefly below and in detail (Technical Appendix).

### CMVRA

The CMVRA (*8*) calculated risk on the basis of average daily temperature, mosquito abundance and infection, counts of WNV RNA–positive dead birds, and sentinel chicken seroconversions over successive 2-week periods. Each variable was assigned to quintile ranks, and these categorical values were averaged to calculate a final risk estimate. Thresholds <2.5 were considered low-risk (normal season) conditions; those 2.6–4.0 were considered medium-risk (emergency planning) conditions; and those >4.1 were considered high-risk (epidemic) conditions.

Details of sampling, laboratory testing, and risk calculation are summarized in the Technical Appendix. In the current study, temperature data were aggregated from the National Aeronautics and Space Administration Terrestrial Observation and Prediction System (*16*) at a 1-km^2^ scale for the GLACVCD jurisdiction. Abundance anomalies for *Culex pipiens quinquefasciatus* mosquitoes collected by gravid traps (*6*) were calculated by comparing current 2-week estimates to 5-year averages for the same period. WNV infection incidence in *Cx. p. quinquefasciatus* mosquitoes was calculated from mosquito pool data by using the Excel (Microsoft, Redmond, WA, USA) add-in developed by Biggerstaff (*17*). Dead birds reported by the public and testing positive for WNV RNA and sentinel chicken seroconversions were ranked according to frequency and scale of occurrence for the broad region (Los Angeles County) and the specific region (within GLACVCD jurisdiction). Reports of sentinel chicken seroconversions from Los Angeles County outside the GLACVCD boundary were found on the California West Nile virus Web site (www.westnile.ca.gov). Human cases, recorded by the Los Angeles County Department of Public Health, Acute Communicable Disease Control, were excluded from the current risk calculations because they were used as an outcome measure.

### Vector Index

The vector index also was calculated for 2-week time steps by using abundance (numbers per gravid trap per night) and infection incidence for *Cx. p. quinquefasciatus* mosquitoes collected by gravid traps by using the bias-corrected maximum-likelihood estimate (*6*) (Technical Appendix). Usually the species-specific maximum-likelihood estimate is multiplied by female mosquito abundance measured by CO_2_ trap counts to yield an arbovirus equivalent of the entomologic inoculation rate in malaria epidemiology (*18*). Vector index estimates were stratified into frequency percentiles by using SAS version 9.1 software (SAS Institute Inc., Cary NC, USA), and the percentiles were assessed individually for their efficacy for predicting human cases.

### DYCAST

For DYCAST, 0.44-km^2^ grid cells were overlaid onto the Los Angeles County study area. There were 22,687 grid cells in Los Angeles County, but only 6,666 grid cells were within the GLACVCD boundary. We assessed the DYCAST risk estimates by using a predetermined Knox test significance threshold of ≤0.10 = high risk. The Knox test statistically delineated significantly positive groups of grid cells into clusters or hot spots. Unlike the other 2 methods, the DYCAST model assessed risk on a daily basis, providing a time and location of high risk on the basis of the spatial grouping of the number of reports of dead birds; data were independent of a predetermined spatial allocation of sampling assets and laboratory diagnostics. To make this method comparable with the previous 2 methods, we selected the minimum DYCAST value by grid for each 2-week period. The DYCAST model then was assessed by using daily and 2-week aggregations.

Another unique feature of the DYCAST model is the spatial resolution. The other 2 methods provide an assessment of high-risk conditions that can be anywhere within the GLACVCD boundary, whereas DYCAST delineates high-risk conditions within a defined space. Again, to make our assessments comparable, we aggregated DYCAST high- and low-risk cells spatially by week, up to the spatial limit imposed by the GLACVCD boundary (6,666 cells). The new spatial aggregates were compared with human case occurrence to determine an optimal number of grid cells needed to establish a high-risk area. This comparison was performed by constructing a receiver operator characteristic (ROC) curve of the plotted sensitivity versus 1 – specificity for all aggregated cell counts.

### Reports of Human Cases

Reports of laboratory-confirmed human cases, compiled by the Acute Communicable Disease Control program of the Los Angeles County Department of Public Health and occurring within GLACVCD, included West Nile fever (WNF) and West Nile neuroinvasive disease (WNND) diagnoses and asymptomatic viremic blood donors. Onset dates for symptomatic persons were adjusted backward 10 days to account for the intrinsic incubation period (*19**,**20*). Seven blood donors with viremia later became symptomatic for WNV disease and were added as WNV cases; the mean time from donation to symptom onset was 6.2 days (SD ±6.14, median 3.5). To account for earlier detection, the infection dates for all viremic blood donors were adjusted backward 4 days (10 latent days minus 6 induction days). As reported for Los Angeles County (*6*), the percentage of WNND among all reported WNV infections increased significantly over time because of reduced physician requests for laboratory testing for febrile illness, thereby reducing the total number of human cases reported. In addition, unpublished data from elsewhere in California also indicate that relatively few persons hospitalized with neuroinvasive disease are tested for WNV, which possibly further reduced recent estimates of WNV-associated human disease. Because mosquito and public health agencies respond to reports of human cases regardless of diagnosis, we chose to use these data in the current analyses. The Institutional Review Board at the University of California, Davis, approved protocols for using human surveillance data (approval no. 201018171-1).

### Analysis

Time-series graphs of the CMVRA and the vector index were plotted with human cases to depict which attained high-risk thresholds before human cases occurred. A true or false-positive finding was a time period identified as high risk during which >1 or 0 human infections occurred, respectively. A true-negative period was a period identified as low risk and during which no human infections occurred; conversely, false-negative periods were identified as low-risk periods when human infections occurred. Sensitivity was calculated as the proportion of high-risk periods correctly identified; specificity was the proportion of low-risk periods correctly identified (*21*). The PPV, likelihood ratio positive, and likelihood ratio negative were calculated as measures of relative precision (*22*).

ROC curves were plotted to define optimum response thresholds. The area under the curve (AUC) was calculated to compare the 2 methods. ROC and AUC calculations were performed by using SAS version 9.1 and the Macro %ROC (http://support.sas.com/kb/25/addl/fusion25017_5_roc.sas.txt).

With the above analyses providing information about the accuracy of each risk assessment, a separate case-crossover study was performed by using the known onset information to create an estimate of the relative risk of acquiring WNV during high-risk periods. Illness onset dates for case-patients and asymptomatic viremic blood donors were lagged backward as described above. Mantel-Haenszel relative risks were calculated to determine whether high-risk values were significantly associated with human infection (*23**–**25*). Mantel-Haenszel relative risks were calculated by using the proportion of high-risk periods before estimated infection as the expected frequency of exposure and the concordance odds of disease transmission occurring during a high-risk period by each model and threshold. Data aggregation and zonal statistics were performed by using PostgreSQL 8.3.7 and PostGIS 1.3.1.

## Results

### CMVRA

**Figure 1:**
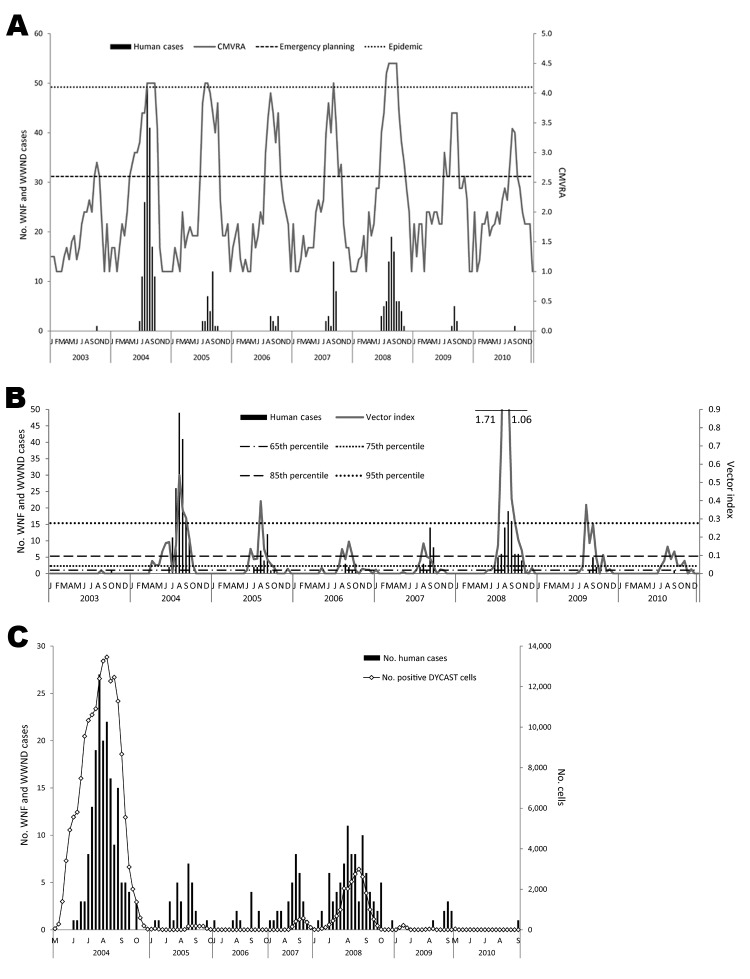
Comparison of risk estimates to human case occurrence for WNF and WWND, Los Angeles, California, USA. A) CMVRA estimates by using *Culex pipiens quinquefasciatus* mosquitoes collected by gravid traps. Dashed lines refer to the risk assessment thresholds of emergency planning at 2.6 and epidemic at 4.1. B) Vector index calculated with *Cx. p. quinquefasciatus* collected by gravid traps. Lines show risk levels discussed in text. C) Weekly counts of positive DYCAST grid cells compared with human case counts. WNF, West Nile fever; WWND, West Nile neuroinvasive disease; CMVRA, California Mosquito-Borne Virus Risk Assessment; DYCAST, Dynamic Continuous-Area Space-Time system.

Risk estimates (Figure 1, panel A) consistently reached emergency planning thresholds (threshold >2.6) before human case detection. In 2004, epidemic thresholds (>4.1) were reached by mid-August (Table 1) after 39 human cases had been reported. During the second epidemic in 2008, risk assessments reached epidemic thresholds after 8 human cases were identified. Using the epidemic threshold, we identified 13 true-positive intervals, 0 false-positive intervals, 151 true-negative intervals, and 28 false-negative intervals. Estimates using this method were driven by ranks for environmental conditions and infections in dead birds, followed by mosquito infection rates and abundance. Antecedent sentinel chicken seroconversions consistently ranked lowest on the 5-point scale until human cases occurred because they were temporally concordant (*26*).

**Table 1:** First dates for risk assessment thresholds and onset of human West Nile disease, Los Angeles, California, USA, 2004–2010*

Model	Threshold	Year	Date	
			Threshold met	First case
CMVRA	2.6, emergency planning	2004	Apr 30	Jun 21
		2005	Jun 30	Jul 5
		2006	Jul 31	Jul 10
		2007	Jul 15	Jul 20
		2008	Jun 15	Jun 24
		2009	Jul 15	Aug 18
		2010	Jun 30	Sep 14
	4.1, epidemic	2004	Aug 15	Jun 21
		2005	Jul 31	Jul 5
		2006	Aug 31	Jul 10
		2007	Sep 15	Jul 20
		2008	Jul 31	Jun 24
		2009	Not observed	Aug 18
		2010	Not observed	Sep 14
Vector index	>0.018, 65th percentile	2004	Apr 15	Jun 21
		2005	Jun 15	Jul 5
		2006	May 15	Jul 10
		2007	May 15	Jul 20
		2008	May 30	Jun 24
		2009	Jul 15	Aug 18
		2010	Jul 15	Sep 14
	>0.069, 80th percentile	2004	Apr 15	Jun 21
		2005	Jun 30	Jul 5
		2006	Aug 15	Jul 10
		2007	Jul 31	Jul 20
		2008	Jul 15	Jun 24
		2009	Aug 15	Aug 18
		2010	Jul 31	Sep 14
DYCAST	Daily	2004	May 4	Jun 21
		2005	Jun 12	Jul 5
		2006	Oct 4	Jul 10
		2007	Aug 13	Jul 20
		2008	Jun 4	Jun 24
		2009	Jun 20	Aug 18
		2010	Apr 5	Sep 14
	Weekly, wk. no.	2004	18	26
		2005	24	28
		2006	40	28
		2007	33	29
		2008	23	26
		2009	24	34
		2010	19	37

*CMVRA, California Mosquito-Borne Risk Assessment; DYCAST, Dynamic Continuous-Area Space-Time system.

Using the emergency planning threshold, we identified 40 true-positive intervals, 28 false-positive intervals, 123 true-negative intervals, and 1 false-negative interval. Although there were more false-positive intervals, they represented high-risk periods before the onset of human cases because the threshold reached >2.6 at least 2 weeks before human cases occurred in all study years except 2008 (Table 1). On the basis of the advance warning that this risk estimate provided and the increase in sensitivity (Table 2), the 2.6 threshold was a better threshold for epidemic prediction.

**Table 2:** Comparison of CMVRA, vector index, and DYCAST for predicting risk for West Nile disease by the calculation threshold applied, validation method, and associated risk, Los Angeles, California, USA, 2004–2010*

Model	Sensitivity	Specificity	PPV	LRP	LRN	Mantel-Haenszel RR (95% CI)
CMVRA						
2.6	0.976	0.815	0.588	5.261	0.03	403.453 (70.506–2,308.659)
4.1	0.317	1	1	UND	0.683	38.255 (29.425–49.736)
Vector index (percentile)						
0.018 (65)	0.974	0.758	0.507	4.029	0.034	25.251 (18.120–35.033)
0.041 (75)	0.846	0.902	0.688	8.631	0.171	25.383 (18.350–35.112)
0.095 (85)	0.564	0.954	0.759	12.33	0.457	24.284 (17.503–33.692)
0.276 (95)	0.246	0.993	0.909	36.231	0.748	23.253 (16.878–32.036)
DYCAST						
Daily	0.268	0.165	<0.001	0.321	4.443	10.112 (7.367–13.880)
Biweekly	0.361	0.045	0.006	0.378	14.242	9.756 (7.764–12.258)

*CMVRA, California Mosquito-Borne Virus Risk Assessment; DYCAST, Dynamic Continuous-Area Space-Time system; PPV, positive predictive value; LRP, likelihood ratio positive; LRN, likelihood ratio negative; RR, relative risk; UND, undefined due to the high specificity.

We calculated sensitivity and specificity separately for each study year by using the 2.6 emergency planning threshold (Table 3). Use of this test validity revealed that sensitivity, i.e., correctly identified high-risk periods, dipped in 2005, whereas specificity, i.e., proportion of correctly identified low-risk periods, was lowest in 2006 and 2008.

**Table 3:** Comparison of the sensitivity and specificity of CMVRA calculated at the emergency planning threshold of 2.6, the vector index calculated at the 80th percentile, and DYCAST risk estimates aggregated weekly for detecting risk for West Nile disease, Los Angeles, California, USA*

Model	2004			2005			2006			2007			2008			2009			2010	
	Sen	Spe		Sen	Spe		Sen	Spe		Sen	Spe		Sen	Spe		Sen	Spe		Sen	Spe
CMVRA	1	0.667		0.857	0.647		1	0.556		1	0.778		0.9	0.571		1	0.857		1	0.913
Vector index	0.778	0.867		0.714	0.941		0.667	1		0.5	1		0.8	1		1	0.714		1	0.652
DYCAST	0.517	0.268		0.034	0.143		0	0		0.063	0		0	0.013		0	0		0	0

*CMVRA, California Mosquito-borne Virus Risk Assessment; DYCAST, Dynamic Continuous-Area Space-Time system; sen, sensitivity; spe, specificity.

### Vector Index

Vector index estimates (Figure 1, panel B) were calculated biweekly for the entire study period for *Cx. p. quinquefasciatus* mosquito collections and were driven exclusively by mosquito infection incidence. Using the 65th percentile (0.018) as the threshold, we identified 38 true-positive, 37 false-positive, 116 true-negative, and 1 false-negative intervals. The frequency distribution of the vector index was highly right skewed and could not be evaluated at lower percentiles because all other percentiles were 0. The vector index increased and remained >0.095 (85th percentile) 4 weeks before the onset of human cases in 2004, 2009, and 2010 and 2 weeks before case onset in 2005 and 2006 (Figure 1, panel B). The sensitivity and specificity of the vector index, calculated annually (Table 3), demonstrated that sensitivity was lower than for the CMVRA in all study years except 2009 and 2010, with the lowest value (0.500) in 2007. The specificity of the vector index was consistently better than that of the CMVRA, except for 2009 and 2010, when only 2 human cases occurred.

### DYCAST

Positive DYCAST cells were observed before human case occurrence in 5 of the 7 study years (Table 1). Counts of positive DYCAST grid cells compared with human case onset is presented in Figure 1, panel C. The DYCAST risk estimate, calculated by grouping the biweekly estimates, was used in the yearly comparisons of sensitivity and specificity (Table 3). Temporal changes in sensitivity and specificity showed the impact of reduced reporting of dead birds over time because the values for both measures of validity were highest in 2004 and declined to 0 or near 0 in all subsequent years.

### Human Case Reports

A total of 389 cases of WNV disease were reported during the study period. Of these, 14 reports were missing onset date information and were not used to evaluate the risk estimates.

### Analysis

The proportion of high-risk intervals correctly identified (sensitivity) was greatest in the CMVRA when the 2.6 emergency planning threshold was used (Table 2). The vector index provided the second highest sensitivity by using values just >0 (65th percentile). The greatest specificity, i.e., proportion of low-risk intervals correctly identified, was observed in the CMVRA at the epidemic threshold of 4.1, followed by the vector index at the 95th percentile; the PPV followed this finding. The likelihood ratio positive, i.e., the likelihood that a high-risk condition was identified correctly when a human case occurred, was greatest for the vector index at the 95th percentile. The likelihood ratio negative, i.e., how much the odds of a human case decrease during low-risk conditions, was lowest in the emergency planning threshold of the CMVRA.

**Figure 2:**
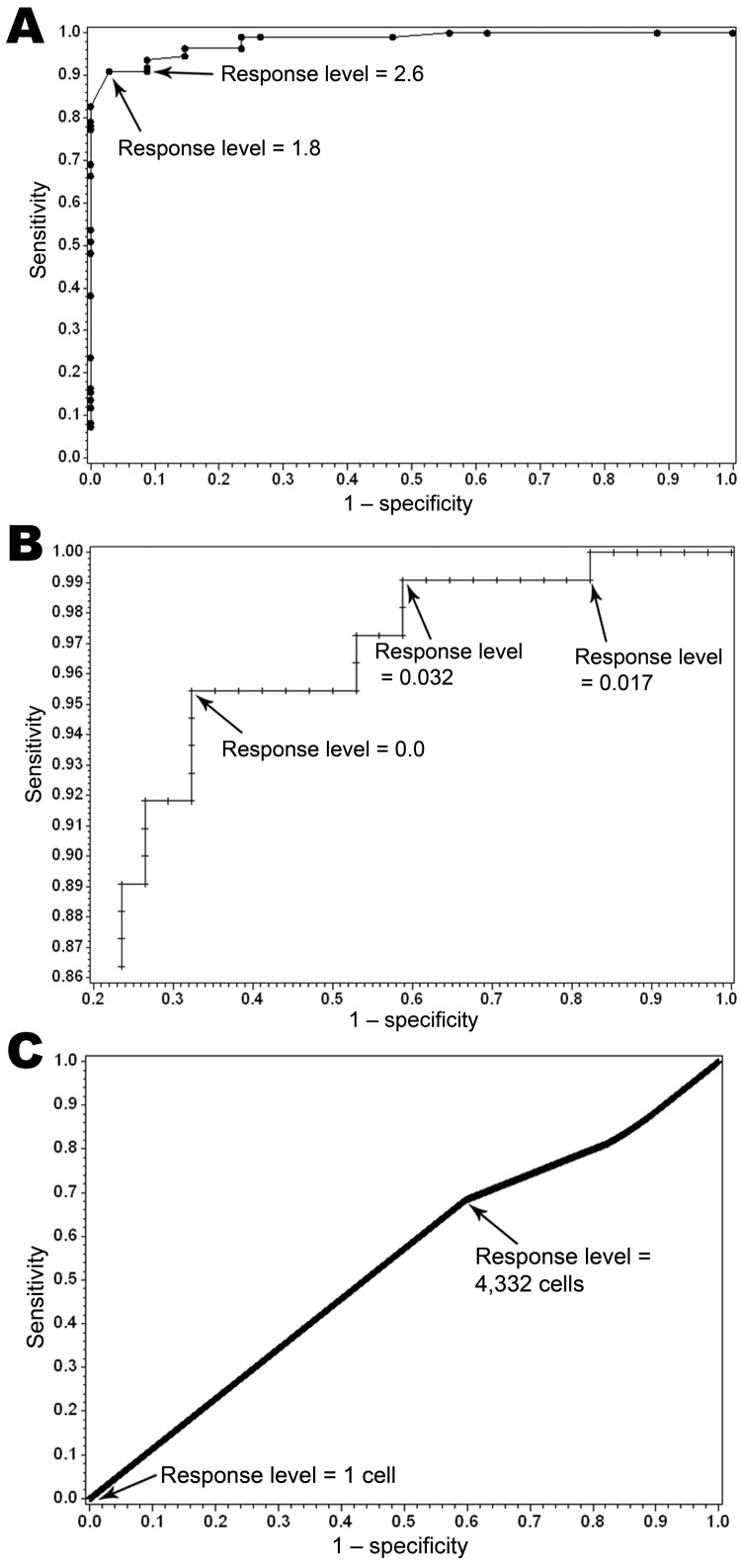
Receiver operator characteristic curves for California Mosquito-Borne Virus Risk Assessment (A), vector index (B), and Dynamic Continuous-Area Space-Time system (C), with labeled cutoff points for 2004–2008 data, Los Angeles, California, USA.

Discriminatory ability, as measured by the AUC, was greatest for the CMVRA (0.982), followed by the vector index (0.845) (Figure 2). Ideal response level cutoffs for the CMVRA as indicated in the ROC plots would be 1.8 and 2.6. The ideal response level for the vector index was more difficult to identify because of the obvious tradeoff between the sensitivity and specificity as evidenced in the ROC plot. The DYCAST cell aggregates performed no better than chance with an AUC of 0.468, with worst performance occurring when a single positive cell was used to assess risk.

A case-crossover study was conducted for all cases and asymptomatic blood donors with a known illness onset or donation date. The relative risk, i.e., risk for WNV infection given exposure to high-risk conditions, was greatest when detected by the CMVRA by using the emergency planning threshold (Table 2).

## Discussion

Since the introduction of WNV into the United States in 1999, WNF and WNND have caused at least 31,365 illnesses and 1,250 deaths (*27*). Once considered to be a mild influenza-like illness, WNF is now understood to be an acute viral infection, often followed by months of illness associated with depression, altered moods, headaches, and fatigue (*28**–**30*). The illness associated with WNND, including meningitis, encephalitis, and acute flaccid paralysis, has been associated with persistent motor and cognitive deficits and incomplete recovery (*28**,**31*). Reported cases of WNF and WNND underrepresent the actual number of WNV cases in the U.S. population (*32**,**33*), and symptomatic persons represent only a fraction of those infected. In addition to individual suffering, the medical and public health costs associated with WNV average >$40,000 per case (*34*) at a time when many health agencies are facing serious budgetary shortfalls.

The individual health toll and associated medical costs present a strong case for active intervention. Current means to prevent WNV infection include integrated vector management by larval mosquito control to arrest viral amplification and, in an outbreak, ground or aerial adulticide applications to eliminate infectious female mosquitoes and personal protection to avoid mosquito bites. Emergency application of adulticides became particularly controversial in California (*35*), even though it is the only method that targets mosquitoes capable of transmitting virus and is cost-effective for preventing human cases (*36*). In light of this controversy, mosquito control agencies in California are often hesitant to apply adulticides until epidemics appear imminent on the basis of available risk estimates or the occurrence of human cases. Our study comparatively evaluated 3 risk measures currently used as decision support tools for intervention and for predicting human cases.

By using only indicators of enzootic transmission, the CMVRA consistently produced estimates in the emergency planning range before human case occurrence; however, epidemic thresholds were not reached until after human cases had been detected. Risk assessment by this method required a robust arboviral surveillance program, with regular sampling for multiple surveillance indicators. The specificity and PPV when the epidemic threshold of 4.1 was used were excellent; however, this was at the expense of adequate lead time for initiating intervention efforts before some human cases. Additionally, the sensitivity of the risk estimate was less than desirable at 0.317, meaning that fewer than one third of the high-risk periods were correctly identified. The CMVRA using the 4.1 threshold was poor at predicting high-risk intervals but good at predicting low-risk intervals.

The 2.6 emergency planning threshold for the CMVRA increased sensitivity and provided a predictive indication of human cases before their onset. The likelihood ratio positive was better than the DYCAST risk estimates, and the likelihood ratio negative was the best of all methods. In addition, the associated risk for human cases, measured by the Mantel-Haenszel relative risk, was the greatest. At the 2.6 emergency planning threshold, the CMVRA was excellent at predicting high-risk periods and good at predicting low-risk periods.

The vector index was simple to calculate and required only a mosquito surveillance and testing program, thereby saving costs associated with sentinel chicken maintenance and sampling and dead bird reporting and testing programs. Unfortunately, this measure did not have preestablished risk thresholds. In our study, it appeared that setting the threshold to >0 (i.e., whenever mosquito infection was detected) would be adequate for predicting human cases in urban settings, such as Los Angeles, where *Cx. p. quinquefaciatus* mosquitoes are the primary vectors and temperatures generally permit viral amplification. The estimates of the vector index increased before case occurrence in 5 of the 7 years. The sensitivity and specificity were comparable with those of the CMVRA, but the likelihood ratio positive was the greatest of all risk estimates. The likelihood ratio negative was better than that of the DYCAST but not as good as that of the CMVRA. Therefore, the vector index was moderate at predicting high-risk periods and very good at predicting low-risk periods. The measure of risk associated with a high-risk value, assessed by the Mantel-Haenszel relative risk, was also better than the DYCAST risk estimate but not as good as either CMVRA threshold.

The DYCAST risk estimate was useful in years with amplified enzootic transmission, when dead birds were considered the primary WNV surveillance indicator (*4**,**37**–**39*). However, after the initial epidemic, WNV activity has been progressively more difficult to predict by using DYCAST because of reduced reporting to the California Dead Bird Hotline. Whether this decrease resulted from truly decreased numbers of dead birds as bird populations became progressively more resistant to infection or to public apathy/decreased awareness was not possible to ascertain. Losing time precision by aggregating estimates clearly increased measures of validity, which considering the uncertainty regarding time between WNV exposure and disease onset seemed appropriate to improve predictive power. The sensitivity of the weekly DYCAST risk estimate was similar to that of the CMVRA, but the specificity, PPV, likelihood ratio positive, and likelihood ratio negative were all uniformly worse than the other 2 methods, even when aggregated spatially. Additionally, the measure of relative risk associated with risk estimates was less than that of the CMVRA and the vector index.

In conclusion, critical decisions on intervention by using risk estimates require knowledge of the strengths and weaknesses of the selected method to respond in an adequate and timely manner to prevent human cases while reducing unnecessary response and costs associated with falsely identified high-risk periods. The goals we set for a good WNV risk estimate were a balance of these attributes and were achieved best in urban and suburban Los Angeles by the CMVRA by using the 2.6 epidemic planning threshold. In light of this finding, an evaluation of the CMVRA should be done in other ecologic settings with transmission driven by other vector species to determine whether the threshold should be adjusted to provide better antecedent estimates of human risk.

Technical Appendix Details of data collection, analysis, and calculation of risk for West Nile virus infection by using the California Mosquito-Borne Virus Surveillance and Response Plan Assessment.

## References

[1] Kramer LD, Styer LM, Ebel GD. A global perspective on the epidemiology of West Nile virus. Annu Rev Entomol. 2008;53:61–81. 10.1146/annurev.ento.53.103106.093258 17645411

[2] Wilson J, Hazelrigg JE, Reisen WK, Madon MB. Invasion of greater Los Angeles by West Nile virus—2003. Proc Mosq Vector Control Assoc Calif. 2004;72:6–11.

[3] Reisen W, Lothrop H, Chiles R, Madon M, Cossen C, Woods L, West Nile virus in California. Emerg Infect Dis. 2004;10:1369–78. 10.3201/eid1008.040077 15496236 PMC3320391

[4] Reisen WK, Barker CM, Carney R, Lothrop HD, Wheeler SS, Wilson JL, Role of corvids in epidemiology of West Nile virus in southern California. J Med Entomol. 2006;43:356–67. 10.1603/0022-2585(2006)043[0356:ROCIEO]2.0.CO;2 16619622

[5] Reisen WK, Fang Y, Lothrop HD, Martinez VM, Wilson J, O’Connor P, Overwintering of West Nile virus in southern California. J Med Entomol. 2006;43:344–55. 10.1603/0022-2585(2006)043[0344:OOWNVI]2.0.CO;2 16619621

[6] Kwan JL, Kluh S, Madon MB, Reisen WK. West Nile virus emergence and persistence in Los Angeles, California, 2003–2008. Am J Trop Med Hyg. 2010;83:400–12. 10.4269/ajtmh.2010.10-0076 20682890 PMC2911193

[7] Ward MR, Stallknecht DE, Willis J, Conroy MJ, Davidson WR. Wild bird mortality and West Nile virus surveillance: biases associated with detection, reporting, and carcass persistence. J Wildl Dis. 2006;42:92–106. 16699152 10.7589/0090-3558-42.1.92

[8] California Department of Public Health, Mosquito and Vector Control Association of California, University of California. California mosquito-borne virus surveillance and response plan. 2009 2009 [cited 2009 Aug 19]. http://westnile.ca.gov/downloads.php?download_id=820&filename=2008_CA_Mosq_Surv.pdf

[9] Barker CM, Reisen WK, Kramer VL. California state mosquito-borne virus surveillance and response plan: a retrospective evaluation using conditional simulations. Am J Trop Med Hyg. 2003;68:508–18. 12812335 10.4269/ajtmh.2003.68.508

[10] Reisen WK, Carroll BD, Takahashi R, Fang Y, Garcia S, Martinez VM, Repeated West Nile virus epidemic transmission in Kern County, California, 2004–2007. J Med Entomol. 2009;46:139–57. 10.1603/033.046.0118 19198528 PMC2729460

[11] Reisen WK, Takahashi RM, Carroll BD, Quiring R. Delinquent mortgages, neglected swimming pools, and West Nile virus, California. Emerg Infect Dis. 2008;14:1747–9. 10.3201/eid1411.080719 18976560 PMC2630753

[12] Barker CM, Bolling BG, Black WC, Moore CG, Eisen L. Mosquitoes and West Nile virus along a river corridor from prairie to montane habitats in eastern Colorado. J Vector Ecol. 2009;34:276–93. 20836831 10.1111/j.1948-7134.2009.00036.x

[13] Bolling BG, Barker CM, Moore CG, Pape WJ, Eisen L. Seasonal patterns for entomological measures of risk for exposure to *Culex* vectors and West Nile virus in relation to human disease cases in northeastern Colorado. J Med Entomol. 2009;46:1519–31. 10.1603/033.046.0641 19960707 PMC2802831

[14] Theophilides CN, Ahearn SC, Grady S, Merlino M. Identifying West Nile virus risk areas: the dynamic continuous-area space–time system. Am J Epidemiol. 2003;157:843–54. 10.1093/aje/kwg046 12727678

[15] Theophilides CN, Ahearn SC, Binkowski ES, Paul WS, Gibbs K. First evidence of West Nile virus amplification and relationship to human infections. Int J Geogr Inf Sci. 2006;20:103–15. 10.1080/13658810500286968

[16] Melton F, Nemani RR, Michaelis A, Barker CM, Park B, Reisen WK. Monitoring and modeling environmental conditions related to mosquito abundance and virus transmission risk with the NASA Terrestrial Observation and Prediction System. Proc Mosq Vector Control Assoc Calif. 2008;76:4.

[17] Biggerstaff BJ. Pooled infection rate. Fort Collins (CO): Centers for Disease Control and Prevention; 2003.

[18] Service MW. Mosquito ecology field sampling methods. London: Applied Science Publishers, Ltd.; 1976.

[19] Centers for Disease Control and Prevention. Transfusion-associated transmission of West Nile virus—Arizona, 2004. MMWR Morb Mortal Wkly Rep. 2004;53:842–4. 15371966

[20] Centers for Disease Control and Prevention. Update: West Nile virus screening of blood donations and transfusion-associated transmission—United States, 2003. MMWR Morb Mortal Wkly Rep. 2004;53:281–4. 15071426

[21] Szklo MaFN. Epidemiology: beyond the basics, 2nd ed. Sudbury (MA): Jones and Bartlett Publishers; 2007.

[22] Johnson KM. The two by two diagram: a graphical truth table. J Clin Epidemiol. 1999;52:1073–82. 10.1016/S0895-4356(99)00087-6 10527001

[23] Dixon KE. A comparison of case–crossover and case–control designs in a study of risk factors for hemorrhagic fever with renal syndrome. Epidemiology. 1997;8:243–6. 10.1097/00001648-199705000-00003 9115017

[24] Maclure M. The case–crossover design—a method for studying transient effects on the risk of acute events. Am J Epidemiol. 1991;133:144–53. 1985444 10.1093/oxfordjournals.aje.a115853

[25] Maclure M, Mittleman MA. Should we use a case–crossover design? Annu Rev Public Health. 2000;21:193–221. 10.1146/annurev.publhealth.21.1.193 10884952

[26] Kwan JL, Kluh S, Madon MB, Nguyen DV, Barker CM, Reisen WK. Sentinel chicken seroconversions track tangential transmission of West Nile Virus to humans in the greater Los Angeles area of California. Am J Trop Med Hyg. 2010;83:1137–45. 10.4269/ajtmh.2010.10-0078 21036853 PMC2963985

[27] Centers for Disease Control and Prevention. West Nile virus, statistics, surveillance and control. 2010 [cited 2010 Jan 16]. http://www.cdc.gov/ncidod/dvbid/westnile/surv&controlCaseCount09_detailed.htm

[28] Sejvar JJ. The long-term outcomes of human West Nile virus infection. Clin Infect Dis. 2007;44:1617–24. 10.1086/518281 17516407

[29] Klee AL, Maldin B, Edwin B, Poshni I, Mostashari F, Fine A, Long-term prognosis for clinical West Nile virus infection. Emerg Infect Dis. 2004;10:1405–11. 10.3201/eid1008.030879 15496241 PMC3320418

[30] Carson PJ, Konewko P, Wold KS, Mariani P, Goli S, Bergloff P, Long-term clinical and neuropsychological outcomes of West Nile virus infection. Clin Infect Dis. 2006;43:723–30. 10.1086/506939 16912946

[31] Sejvar JJ, Haddad MB, Tierney BC, Campbell GL, Marfin AA, Van Gerpen JA, Neurologic manifestations and outcome of West Nile virus infection. JAMA. 2003;290:511–5. 10.1001/jama.290.4.511 12876094

[32] Busch MP, Wright DJ, Custer B, Tobler LH, Stramer SL, Kleinman SH, West Nile virus infections projected from blood donor screening data, United States, 2003. Emerg Infect Dis. 2006;12:395–402. 10.3201/eid1203.051287 16704775 PMC3291460

[33] Centers for Disease Control and Prevention. West Nile virus: clinical description. 2008 [cited 2010 Jan 16]. http://www.cdc.gov/ncidod/dvbid/westnile/clinicians/clindesc.htm

[34] Zohrabian A, Meltzer M, Ratard R, Billah K, Molinari N, Roy K, West Nile virus economic impact, Louisiana, 2002. Emerg Infect Dis. 2004;10:1736–44. 10.3201/eid1010.030925 15504258 PMC3323281

[35] Peterson RK, Macedo PA, Davis RS. A human-health risk assessment for West Nile virus and insecticides used in mosquito management. Environ Health Perspect. 2006;114:366–72. 10.1289/ehp.8667 16507459 PMC1392230

[36] Barber LM, Shleier JJ, Peterson RKD. Economic cost analysis of West Nile virus outbreak, Sacramento, California, USA, 2005. Emerg Infect Dis. 2010;16:480–6. 10.3201/eid1603.090667 20202424 PMC3322011

[37] Mostashari F, Kulldorff M, Hartman JJ, Miller JR, Kulasekera V. Dead bird clusters as an early warning system for West Nile virus activity_._ Emerg Infect Dis. 2003;9:641–6. 10.3201/eid0906.020794 12781002 PMC3000152

[38] Eidson M, Kramer L, Stone W, Hagiwara Y, Schmit K. Dead bird surveillance as an early warning system for West Nile virus. Emerg Infect Dis. 2001;7:631–5. 11585524 10.3201/eid0704.010405 PMC2631773

[39] Carney RM, Ahearn SC, McConchie A, Glaser C, Jean C, Barker C, Early warning system for West Nile virus risk areas, California, USA. Emerg Infect Dis. 2011;17:1445–54. 21801622 10.3201/eid1708.100411 PMC3381548

