# Supplementary material for: Comparison of Enzootic Risk Measures for Predicting West Nile Disease, Los Angeles, California, USA, 2004–2010
Source: Emerg Infect Dis. 2012 Aug;18(8):1298–306. doi: 10.3201/eid1808.111558 (PMC3414020; doi:10.3201/eid1808.111558)
Supplement: Technical Appendix — Details of data collection, analysis, and calculation of risk for West Nile virus infection by using the California Mosquito-Borne Virus Surveillance and Response Plan Assessment. [file 11-1558_Techapp-s1.pdf]

Article DOI: http://dx.doi.org/10.3201/eid1808.111558

Comparison of Enzootic Risk Measures for Predicting West Nile Disease, Los Angeles, California, USA, 2004–2010

Technical Appendix

Details of Data Collection, Analysis, and Calculation of Risk by Using the CMVRA

The risk calculation form, downloaded from the CMVRA (http://westnile.ca.gov/downloads.php?download_id=2321&filename=2012%20CA%20Response%20Plan%205-8-12.pdf), is shown in Technical Appendix Tables 1 and 2. Each of 5 factors were assigned a risk level increasing from 1 (low) to 5 (high) based on data accumulated by the GLACVCD during the previous 2-week time step. These values then were arithmetically averaged and risk assessed based as "low" or normal season, "epidemic planning" with increasing trends in some/all factors, and "epidemic" with most factors >4. Although each of the 5 factors have their own variance about the estimates, it was not possible to readily combine these into an estimate error about the arithmetic average of the rank values. Standard error calculated for this mean was proportional to increasing ranks and therefore increased with increasing risk. The overall risk model was designed to be adapted to local conditions in a large state with markedly different ecologic conditions, vector populations, and control agency budgets. Data from Los Angeles were gathered by the GLACVCD as described previously (*1*) and detailed in general below:

1. Weather. Because there is little rain during the transmission season, only temperature is considered. Escalating risk is based on the decreasing duration of the extrinsic incubation period of WNV in *Cx. tarsalis* as a function of temperature (*2*), where 14.3°C is the threshold for virus growth. Here, antecedent warm temperatures reduce the age at which vector transmission may occur and defines elevated risk.

2. Vector abundance. There are 2 primary vector species in California, and risk can be calculated separately for each; however, only *Cx. p. quinquefasciatus* is abundant in Los Angeles (*1*), and data here were restricted to this species. Risk was determined by counts of *Cx. p. quinquefasciatus* females in gravid traps (*3*), transformed by ln [y+1] to normalize the distribution, averaged among traps and then backtransformed. These geometric means are compared with means calculated for the same time period over the previous 5 years, expressed as a percentage anomaly, and assigned to a category of escalating risk from 1 to 5 (Technical Appendix Tables 1, 2).

3. Vector infection. Female *Cx. p. quinquefasciatus* from the above traps were pooled into lots of ≤50 females each, stored at −80°C, and shipped on dry ice to the Center for Vectorborne Diseases, where they were tested for WNV RNA by qRT-PCR by using an ABI 7900 platform (Applied Biosystems, Foster City, CA, USA) and primers and probes described by Lanciotti et al. (*4*). Pools also were tested concurrently for WEEV and SLEV, but were negative during the current study period. Infection rates per 1,000 for each 2-week time step were calculated by the bias corrected maximum likelihood estimate [MLE] by using the Excel spreadsheet add-in described by Biggerstaff (*5*) and available from the CDC West Nile virus website. MLE estimates were ranked 1–5 (Technical Appendix Tables 1, 2) and assigned an escalating risk value based on previous field studies in California.

4. Sentinel chickens. Flocks of 7 sentinel hens were deployed at 7 locations throughout the GLACVCD in March–April and then bled at 2-week intervals until replaced the following season or when >5 seroconverted. Serum was tested for evidence of previous WNV infection by an enzyme immunoassay with positives confirmed by Western blot or plaque reduction neutralization test (*6*). Risk was based on the spatial distribution and number of seroconversions detected during the 2-week time step (Technical Appendix Tables 1, 2), with the "broad region" Los Angeles County and the "specific region" the GLACVCD jurisdiction.

5. Dead bird reporting and testing. Many species of California birds die due to WNV (*7*), usually within 5–7 days of infection (*8*,*9*), thereby providing a measure of recent transmission. In Los Angeles, large populations of American crows have suffered severe die-offs and these have been provided a useful measure of WNV activity (*1*,*10*). Dead or dying birds were reported to the California Dead Bird Hotline by the public, collected by GLACVCD personnel, and shipped to the California Animal Health and Food Safety laboratory for necropsy under BSL-3 conditions. Oral swabs and kidney samples then were sent to Center for Vectorborne Diseases where they were tested by qRT-PCR for WNV RNA as described above for mosquitoes. Risk was based on the geographic distribution and numbers of WNV-positive dead birds (Technical Appendix Tables 1, 2).

In the calculation example below, the 2-week average daily temperature was warm (77°F), *Cx. p. quinquefasciatus* females were moderately abundant, averaging 28 females per gravid trap per night (280% above the 5-year average for the same time period), and 10 pools consisting of 50 females each were tested and 3 were WNV positive. The resulting MLE estimate of 6.69 had a broad 95% CI of 1.8–18.8 but was ranked as 5. In addition, 4 chickens in 3 flocks seroconverted, and 8 American crows and 2 house finches tested positive of 26 submitted for testing—all were collected within the GLACVCD boundaries. This resulted in a risk score of 4.4, placing the GLACVCD at epidemic level of risk, indicating an ongoing epidemic and the probable occurrence of human cases.

Technical Appendix Table 1. Risk calculation form

Factor	Factor value	Calculation	Risk score
1. Temp	°	none	4
2. Abundance	28 F/TN	280% = 28/10	4
3. Infection	3 WNV+/10	6.69 (1.8-18.8)	5
4. Sentinels	4 in 3 flocks	none	4
5. Dead birds	10 WNV+	none	5
		Average	4.4

Technical Appendix Table 2. Risk calculation form details

WNV Surveillance Factor	Assessment Value	Benchmark	Assigned Value	
1. Environmental Conditions<br>High-risk environmental conditions include above-normal temperatures with or without above-normal rainfall, runoff, or snowpack. Weather data link: http://ipm.ucdavis.edu	1	Avg daily temperature during prior 2 weeks ≤ 56 °F		
	2	Avg daily temperature during prior 2 weeks 57 – 65 °F		
	3	Avg daily temperature during prior 2 weeks 66 – 72 °F		
	4	Avg daily temperature during prior 2 weeks 73 – 79 °F		
	5	Avg daily temperature during prior 2 weeks > 79 ° F		
			Cx tars	*Cx pip*
2. Adult *Culex tarsalis* and *Cx. pipiens* complex relative abundance*<br>Determined by trapping adults, enumerating them by species, and comparing numbers to those previously documented for an area for the prior 2-week period.	1	Vector abundance well below average (≤ 50%)		
	2	Vector abundance below average (51 - 90%)		
	3	Vector abundance average (91 - 150%)		
	4	Vector abundance above average (151 - 300%)		
	5	Vector abundance well above average (> 300%)		
3. Virus infection rate in *Culex tarsalis* and *Cx. pipiens* complex mosquitoes*<br>Tested in pools of 50. Test results expressed as minimum infection rate per 1,000 female mosquitoes tested (MIR) for the prior 2-week period.	1	MIR = 0		
	2	MIR = 0.1 - 1.0		
	3	MIR = 1.1 - 2.0		
	4	MIR = 2.1 - 5.0		
	5	MIR > 5.0		
4. Sentinel chicken seroconversion<br>Number of chickens in a flock that develop antibodies to WNV during the prior 2-week period. If more than one flock is present in a region, number of flocks with seropositive chickens is an additional consideration. Typically 10 chickens per flock.	1	No seroconversions in broad region		
	2	One or more seroconversions in broad region		
	3	One or two seroconversions in a single flock in specific region		
	4	More than two seroconversions in a single flock or two flocks with one or two seroconversions in specific region		
	5	More than two seroconversions per flock in multiple flocks in specific region		
5. Dead bird infection<br>Number of birds that have tested positive (recent infections only) for WNV during the prior 3-month period. This longer time period reduces the impact of zip code closures during periods of increased WNV transmission.	1	No positive dead birds in broad region		
	2	One or more positive dead birds in broad region		
	3	One positive dead bird in specific region		
	4	Two to five positive dead birds in specific region		
	5	More than five positive dead birds in specific region		
6. Human cases<br>Do not include this factor in calculations if no cases are detected in region.	3	One or more human infections in broad region		
	4	One human infection in specific region		
	5	More than one human infection in specific region		
			Cx tars	*Cx pip*
<u>Response Level / Average Rating:</u><br>Normal Season (1.0 to 2.5)<br>Emergency Planning (2.6 to 4.0)<br>Epidemic (4.1 to 5.0)		TOTAL		
		AVERAGE		

*Calculation of separate risk values for *Cx. tarsalis* and the *Cx. pipiens* complex may be useful if their spatial distributions (e.g., rural vs. urban) differ within the assessment area.

Vector Index

The vector index calculations used in this paper were developed by Roger Nasci, Research Entomologist for the National Center for Infectious Disease at CDC (www.cdphe.state.co.us/dc/zoonosis/wnv/Nasci_VectorIndexPoster.pdf) (Technical Appendix Table 3). This simple metric multiplies the mosquito infection rate by the mosquito abundance.

As with the CMVRA, the data were aggregated into the same 2-week previous interval for calculation.

Technical Appendix Table 3. Vector index metric

Factor	Factor value
Abundance	28 F/TN
Infection rate	6.69/1,000
Calculation	0.187

DYCAST

The DYCAST estimates were achieved by geocoding dead bird reports for the state of California. A 0.5-mile grid was then superimposed on the state, and the dead bird reports were assigned to the center of each grid cell. Knox space–time interaction tests were performed to determine whether the reported birds were "close" in both dimensions. The Knox test creates pairs of bird reports and assigns a value of 0 if the distance between the 2 reports in the pair is greater than the critical distance, 0.40 km (t_{ij}), (1 if within the critical distance) and a 0 if the time between reports is greater than the critical time of 3 days (s_{ij}) (1 if within). The test statistic is the summation, over all bird pairs, of the products of t_{ij} and s_{ij}, and is compared with a random spatiotemporal distribution of reports. A Knox test p value of >0.1 was considered low risk, and a p value of $\leq$0.10 was considered high risk. The minimum number of birds required for calculation of the statistic was 15 per cell, and the results were calculated daily (*11*).

In our analyses, we assessed the DYCAST daily estimates as well as 2-week aggregates where we selected the minimum p value over the same 2-week periods as the CMVRA and vector index. To assess the spatial accuracy we aggregated high- or low-risk cells up to the limit of the GLACVCD boundary.

References

1. Kwan JL, Kluh S, Madon MB, Reisen WK. West Nile virus emergence and persistence in Los Angeles, California, 2003–2008. Am J Trop Med Hyg. 2010;83:400–12. PubMed http://dx.doi.org/10.4269/ajtmh.2010.10-0076

2. Reisen WK, Fang Y, Martinez VM. Effects of temperature on the transmission of West Nile virus by *Culex tarsalis* (Diptera: Culicidae). J Med Entomol. 2006;43:309–17. PubMed http://dx.doi.org/10.1603/0022-2585(2006)043[0309:EOTOTT]2.0.CO;2

3. Cummings RF. Design and use of a modified Reiter gravid mosquito trap for mosquito-borne encephalitis surveillance in Los Angeles County, California. Proceedings of the Mosquito and Vector Control Association of California. 1992;60:170–6.

4. Lanciotti RS, Kerst AJ. Nucleic acid sequence-based amplification assays for rapid detection of West Nile and St. Louis encephalitis viruses. J Clin Microbiol. 2001;39:4506–13. PubMed http://dx.doi.org/10.1128/JCM.39.12.4506-4513.2001

5. Biggerstaff BJ. Software for mosquito surveillance. Pooled infection rate [cited 2003]. http://www.cdc.gov/ncidod/dvbid/westnile/software.htm

6. Patiris PJ, Oceguera LF III, Peck GW, Chiles RE, Reisen WK, Hanson CV. Serologic diagnosis of West Nile and St. Louis encephalitis virus infections in domestic chickens. Am J Trop Med Hyg. 2008;78:434–41. PubMed

7. Wheeler SS, Barker CM, Armijos MV, Carroll BD, Husted SR, Reisen WK. Differential impact of West Nile virus on California birds. Condor. 2009;111:1–20. PubMed http://dx.doi.org/10.1525/cond.2009.080013

8. Komar N, Langevin S, Hinten S, Nemeth N, Edwards E, Hettler D, et al. Experimental infection of North American birds with the New York 1999 strain of West Nile virus. Emerg Infect Dis. 2003;9:311–22. PubMed

9. Reisen WK, Fang Y, Martinez VM. Avian host and mosquito (Diptera: Culicidae) vector competence determine the efficiency of West Nile and St. Louis encephalitis virus transmission. J Med Entomol. 2005;42:367–75. PubMed http://dx.doi.org/10.1603/0022-2585(2005)042[0367:AHAMDC]2.0.CO;2

10. Carney R, Padgett K, Cahoon-Young B, Woods L, Read D, Reisen WK, et al. The California West Nile virus dead bird surveillance program—challenges and solutions during 2004. Proceedings of the Mosquito and Vector Control Association of California. 2005;73:105–6.

11. Carney RM, Ahearn SC, McConchie A, Glaser C, Jean C, Barker C, et al. Early warning system for West Nile virus risk areas, California, USA. Emerg Infect Dis. 2011;17:1445–54. PubMed
